# Diversity of Both the Cultivable Protease-Producing Bacteria and Bacterial Extracellular Proteases in the Coastal Sediments of King George Island, Antarctica

**DOI:** 10.1371/journal.pone.0079668

**Published:** 2013-11-04

**Authors:** Ming-Yang Zhou, Guang-Long Wang, Dan Li, Dian-Li Zhao, Qi-Long Qin, Xiu-Lan Chen, Bo Chen, Bai-Cheng Zhou, Xi-Ying Zhang, Yu-Zhong Zhang

**Affiliations:** 1 State Key Laboratory of Microbial Technology, Shandong University, Jinan, China; 2 Marine Biotechnology Research Center, Shandong University, Jinan, China; 3 SOA Key Laboratory for Polar Science, Polar Research Institute of China, Shanghai, P. R. China; U.S. Geological Survey, United States of America

## Abstract

Protease-producing bacteria play a vital role in degrading sedimentary organic nitrogen. However, the diversity of these bacteria and their extracellular proteases in most regions remain unknown. In this paper, the diversity of the cultivable protease-producing bacteria and of bacterial extracellular proteases in the sediments of Maxwell Bay, King George Island, Antarctica was investigated. The cultivable protease-producing bacteria reached 10^5^ cells/g in all 8 sediment samples. The cultivated protease-producing bacteria were mainly affiliated with the phyla Actinobacteria, Firmicutes, Bacteroidetes, and Proteobacteria, and the predominant genera were *Bacillus* (22.9%), *Flavobacterium* (21.0%) and *Lacinutrix* (16.2%). Among these strains, *Pseudoalteromonas* and *Flavobacteria* showed relatively high protease production. Inhibitor analysis showed that nearly all the extracellular proteases from the bacteria were serine proteases or metalloproteases. These results begin to address the diversity of protease-producing bacteria and bacterial extracellular proteases in the sediments of the Antarctic Sea.

## Introduction

There is abundant particulate organic material in the sea floor. Bacterial enzymatic activity in sediment is generally considered to be the initial and rate-limiting step in nitrogen recycling [1]. Protease-producing bacteria are important decomposers of sedimentary organic nitrogen (OrgN) because proteins are an important component of the OrgN. However, only a few studies on the diversity of sedimentary protease-producing bacteria and their proteases have been reported. Olivera et al. [2] screened 19 protease-producing strains from sub-Antarctic sediments; they belonged to the genera *Pseudoalteromonas, Shewanella, Colwellia*, and *Planococcus*, and the family Flavobacteriaceae. We screened 78 protease-producing strains from the sediments of the South China Sea and investigated the diversity of both the cultivable protease-producing bacteria and their extracellular proteases [3]. It was found that these protease-producing bacterial strains were mainly affiliated with the class Gammaproteobacteria and that their extracellular proteases were serine proteases and metalloproteases. The global sea floor is widespread with various environments; thus, the protease-producing bacteria and their extracellular proteases in different regions may differ in type and quantity.

The Antarctic continent is surrounded by the Southern Ocean. In coastal regions, the marine productivity was estimated to be upwards of 300 g of C m^-2^ year^-1^ [4]. Antarctic bottom water is quite nutrient rich, contributing to enhanced biological activity in Antarctic coastal regions [5,6]. Investigating the microbial communities and their functions in coastal sediments is vital to elucidating the biogeochemical cycles in the Southern Ocean. There have been some reports aimed at the proteases or protease-producing bacteria in Antarctic marine sediment [7-9]; however, the diversity of protease-producing bacteria in these permanently low-temperature sediments has not been investigated.

The maritime Antarctic region covers the western part of the Antarctic Peninsula and nearby islands. Here the climate is less extreme than on the Antarctic continent, with an average temperature of approximately 0°C during the austral summer. In this paper, 8 coastal sediment samples were collected from Maxwell Bay, King George Island, which is located in the maritime Antarctic region, and the diversity of cultivable protease-producing bacteria and of bacterial extracellular proteases from these sediment samples was investigated.

## Materials and Methods

### Ethics Statement

All 8 sediment samples were collected from the sea adjacent to the King George Island, not from the land. The nearest Antarctic Special Protected Areas (ASPA) to our sample positions are ASPA No.125 and No.150, which do not include the adjacent sea area (http://www.ats.aq/devPH/apa/ep_protected.aspx). Thus, no specific permissions were required for these locations/activities. Additionally, our sediment studies did not involve endangered or protected species.

### Sampling and Geochemical Characteristics of Samples

Sediment samples were collected using a sediment grab sampler during the 26th Chinese Antarctic Research Expedition in 2010. Only undisturbed samples were used to ensure the integrity of the surface sediment structures. Three replicate surface sediment (0-5 cm) subcore samples were taken aseptically with sterile tubes and stored in airtight sterile plastic bags at 4°C for 1-4 days prior to microbiological analysis. Sediment samples for environmental analysis were taken in the same manner and stored in airtight sterile plastic bags at -80°C. Sediment organic carbon (OrgC) and OrgN contents were measured in the laboratory with a PE 2400 Series II CHNS/O analyzer (Perkin Elmer, USA).

### Cultivation and Screening of Protease-Producing Bacteria

Cultivation and screening of protease-producing bacteria were performed as described previously [3]. One gram (wet weight) of sediment from every sample was ten-fold serially diluted to a 10^-6^ dilution with sterile artificial seawater that was prepared by dissolving sea salt (Sigma) in distilled water (salinity, ~35 ppt). Aliquots of 100 µl of diluted sediment samples (10^-1^-10^-6^ dilutions) were spread on screening plates with a medium composed of 0.2% yeast extract, 0.3% casein, 0.5% gelatin, 1.5% agar powder and artificial seawater (pH 8.0). The plates were then incubated at 15°C for 2-4 days to form detectable colonies with a clear hydrolytic zone. Morphologically different colonies with or without hydrolytic zones were selected and further purified by repeatedly streaking on the same medium. The purified strains were stored in 12% glycerol at -80°C.

### Amplification of 16S rRNA Genes and Phylogenetic Analysis

Bacterial 16S rRNA genes were amplified by PCR as described previously with primers 27F and 1492R [3]. These genes were ligated into pMD-18T cloning vectors (TAKARA) and sequenced by the Beijing Genomics Institute (China). Sequence alignment was performed using CLUSTAL X (v 1.83) [10]. Isolates with one or more different bases in their 16S rRNA gene sequences were considered to be different strains. Neighbor-joining trees were constructed using MEGA version 5.05 [11] with the neighbor-joining method and the Kimura two-parameter model. 

### Protease Production of the Strains on Casein-Gelatin Plates

Plates were prepared with the screening medium (pH 8.0) containing casein and gelatin. The strains that produced a hydrolytic zone on the screening plates were streaked on the prepared plates, and incubated at 15°C for 4 d. Then, for each strain, the diameter of its colony and the diameter of the hydrolytic zone it produced were measured, and the ratio of the hydrolytic zone diameter to the colony diameter (hydrolytic zone/colony, H/C) was calculated. 

### Analysis of the Inhibitory Effect of Protease Inhibitors on Protease Activity

 The inhibitor assay was performed as described previously [3]. The protease-producing strains were grown in the liquid screening medium at 15°C at 200 rpm for 4 d. The culture was centrifuged at 12,000 g at 4°C for 10 min. The protease activity of the supernatant was measured as previously described [12]. The supernatant was diluted with 50 mM Tris-HCl (pH 8.0) to maintain an optical density reading at OD_660_ between 0.3-1.0 in subsequent protease activity analysis, and was pre-incubated with 1.0 mM phenylmethylsulfonyl fluoride (PMSF, Sigma), 1.0 mM 1,10-Phenanthroline (OP, Sigma), 0.1 mM E-64 (Merck) or 0.1 mM Pepstatin A (Merck) at 15°C for 20 min. After incubation, the protease activity of every sample was measured by using the method previously described [12]. The activity of a sample without any inhibitor was set to 100% activity, and the relative activity (%) of samples was calculated. The inhibition ratio was taken as the result of the control activity minus the relative activity of a sample. 

### Nucleotide Sequence Accession Numbers

The 16S rRNA gene sequences resulting from this study were deposited in GenBank under the accession numbers KC160632- KC160834.

## Results

### Station description and sample characteristics

**Figure 1:**
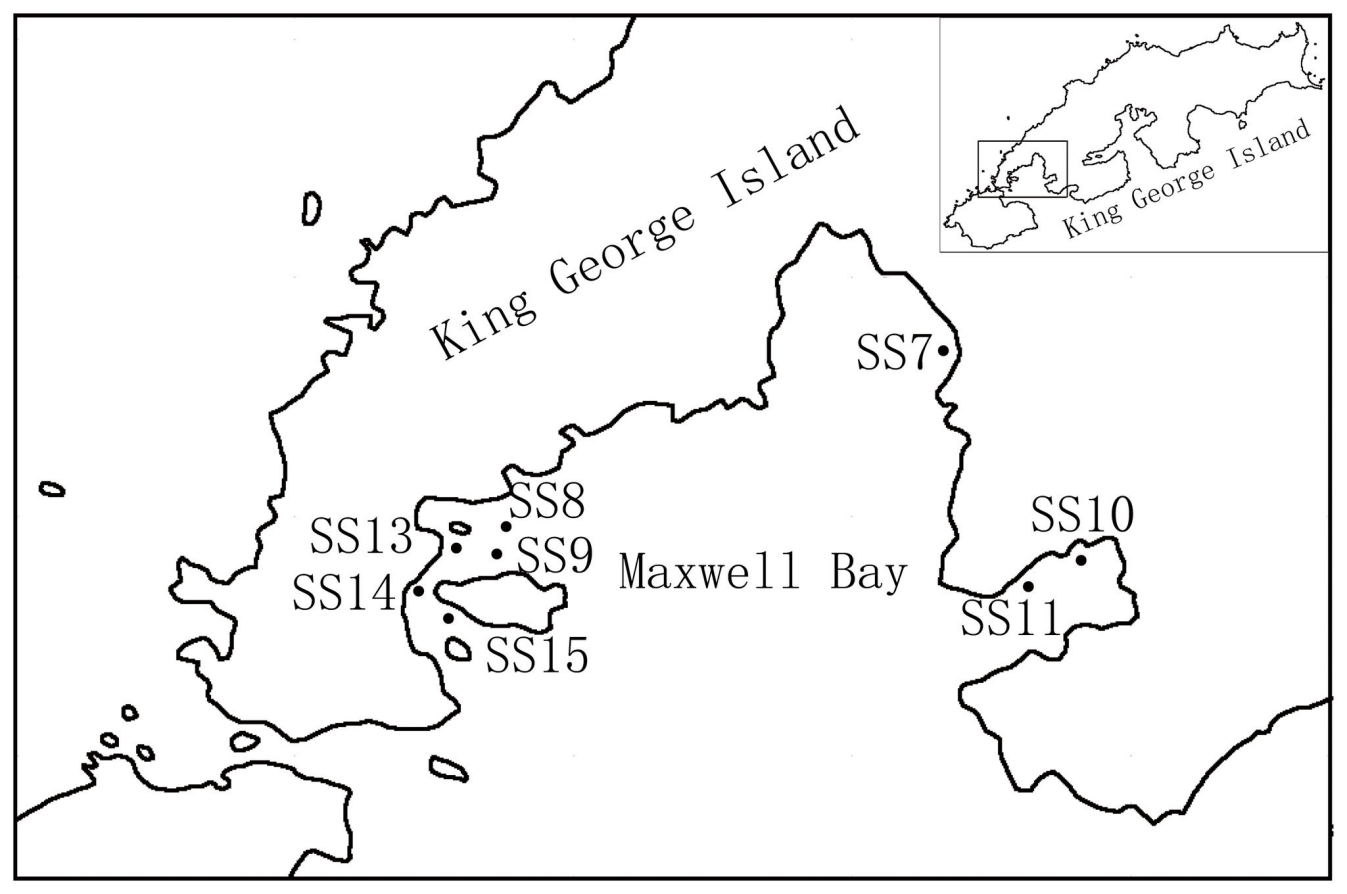
Geographic location of sampled sediment stations in Maxwell Bay, Antarctica.

The sampling stations with variable water depths (15-55 m) are located in different areas of Maxwell Bay, King George Island, Antarctica (Figure 1, Table 1). Stations SS7 and SS10 are located next to Collins Icecap.

**Table 1:** Characteristics of the sampling stations.

Station	Location (S,W)		Depth (m)	OrgC (%)	OrgN (%)	C:N
SS7	62°10’13.42”	58°47’47.54”	35.8	0.63	0.57	1.11
SS8	62°12’12.20”	58°56’20.26”	25	0.56	0.49	1.16
SS9	62°12’21.48”	58°56’48.98”	55	1.02	0.61	1.68
SS10	62°12’10.27”	58°44’50.21”	21	0.32	0.54	0.598
SS11	62°12’19.31”	58°45’41.47”	15	0.41	0.50	0.830
SS13	62°12’28.31”	58°56’41.22”	17	1.69	0.64	2.65
SS14	62°12’38.36”	58°57’11.29”	34	3.59	1.07	3.35
SS15	62°12’51.89”	58°56’59.12”	25	3.40	0.93	3.66

The content of OrgC and OrgN in the sediments was in the range of 0.32-3.59% (OrgC) and 0.49-1.07% (OrgN); the highest values were obtained from stations SS14 and SS15, and the lowest values were obtained from station SS11 (Table 1). The highest C/N ratio was observed at station SS15 (3.66), and the lowest was observed at station SS10 (0.598). 

### Screening of Protease-Producing Bacteria from Sediments

After cultivation on screening plates, colonies appeared on the plates of the 10^-1^ - 10^-4^ diluted samples. Quantitative statistics by manual count showed that the abundance of cultivated bacteria reached 10^5^ cells/g in all sediment samples, and approximately 40% of the colonies produced obvious hydrolytic zones in all samples. The difference of OrgC and OrgN content and of the C/N ratio among the stations (Table 1) did not lead to obvious differences in the richness of cultivable protease-producing bacteria. Two hundred and twenty-four colonies were purified for identification and characterization. 

### Diversity of protease-producing bacteria in the sediments

**Figure 2:**
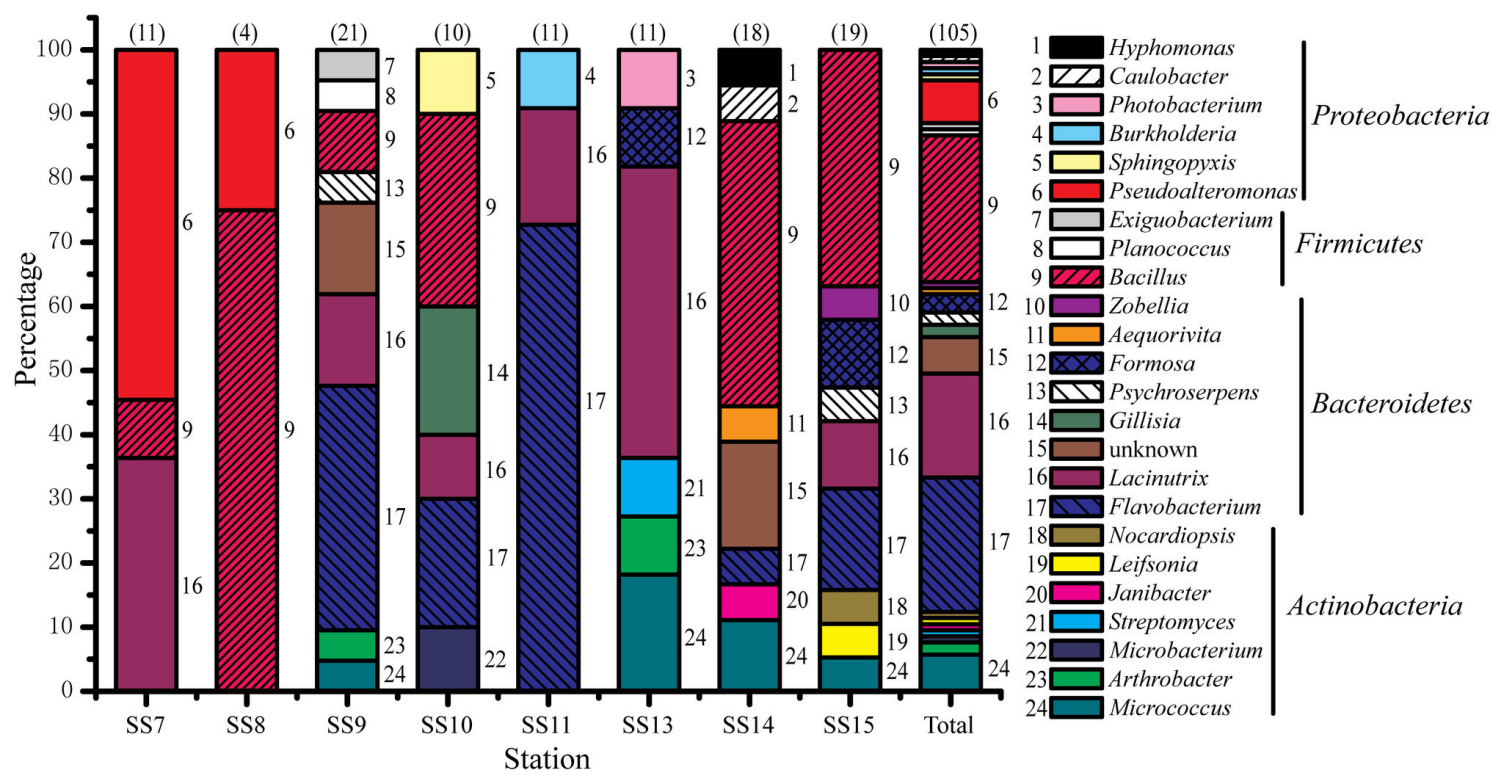
Relative percentage abundances of the phylotypic groups of cultivable protease-producing bacteria recovered from eight sampled stations in Maxwell Bay, Antarctica. The number in parentheses above each bar indicates the total number of sequences it represents. Different genera are indicated by the numbers at the right of each section.

Nearly complete 16S rRNA genes of the 224 isolates were amplified and sequenced. Isolates with identical 16S rRNA gene sequences were considered to be the same strain, and consequently, 203 different strains were identified. Among them, 98 strains could grow on the screening plates but did not form a hydrolytic zone and were not further studied for their slight extracellular protease-producing ability. Based on the 16S rRNA genes sequences, the phylogenetic affiliation of the 105 strains that formed hydrolytic zones on the screening plates was analyzed. In total, 23 genera and 5 unknown Bacteroidetes strains were identified from these samples (Figure 2). The strains were affiliated with the phyla Firmicutes, Bacteroidetes, Actinobacteria and Proteobacteria. In summary, the genera *Bacillus* (22.9%), *Flavobacterium* (21.0%) and *Lacinutrix* (16.2%) were the most cultivated groups. Of the 23 genera identified, 14 were represented by a single strain. We were able to cultivate a greater variety of genera at some stations (SS9, SS14, SS15) compared to others (e.g., SS8). In the sediment sample SS7, the predominant cultivated protease-producing bacteria were *Pseudoalteromonas* (66.7%). In contrast, the predominant cultivated protease-producing bacteria in the other sediment samples were *Bacillus*, *Flavobacterium* or *Lacinutrix*. Only 4 protease-producing bacterial strains were recovered from sample SS8, including 3 *Bacillus* and 1 *Pseudoalteromonas* strains (Figure 2).

**Figure 3:**
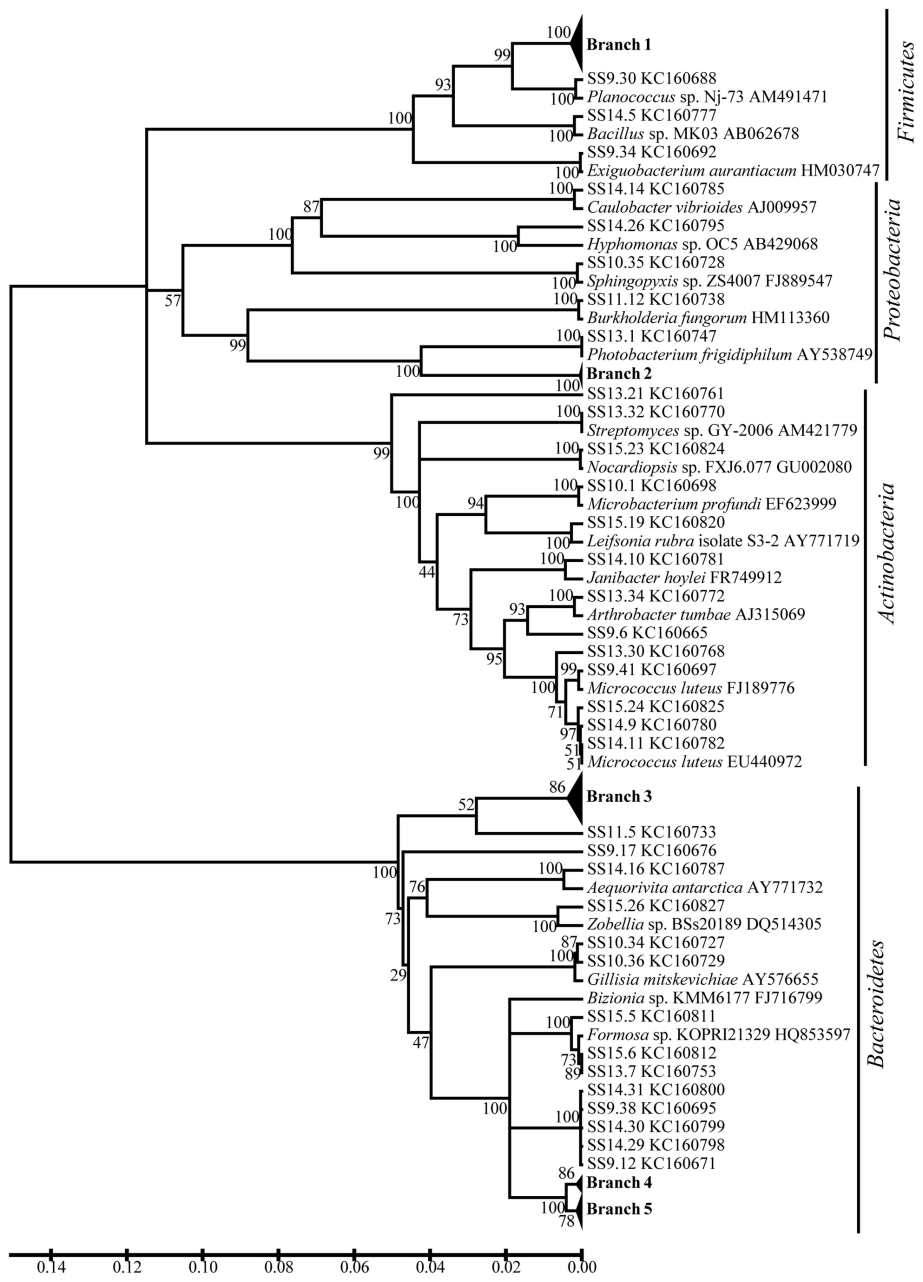
Neighbor-joining phylogenetic tree of the protease-producing bacteria recovered from eight sampled sediment stations in Maxwell Bay, King George Island based on the 16S rDNA sequences. Branch 1 indicates 23 *Bacillus* strains similar to *Bacillus* sp. Nj-19 (AM491453). Branch 2 indicates 7 *Pseudoalteromonas* strains similar to *Pseudoalteromonas arctica* (DQ787199). Branch 3 indicates 21 *Flavobacterium* strains similar to *Flavobacterium degerlachei* (AJ557886) and *Flavobacterium degerlachei* (EU000231). Branch 4 indicates 6 *Psychroserpens* strains similar to *Psychroserpens* sp. A622 (AY781191). Branch 5 indicates 13 *Lacinutrix* strains similar to *Lacinutrix* sp. E4-9a (FN377744). The neighbor-joining phylogenetic trees of strains in Branch 1, 2, 3, 4 and 5 based on the 16S rDNA sequences are shown in Figures S1, S2, S3, S4 and S5, respectively.

A distance-based neighbor-joining tree was constructed using the sequences from this study and reference sequences from the GenBank database (Figure 3). Strains related to *Bacillus*, *Lacinutrix* and *Flavobacterium* were the most frequently recovered isolates (recovered from 6, 6 and 5 sediments, respectively) and formed the largest groups in term of abundance (63 of 105 strains). Twenty-four *Bacillus* strains recovered from 6 sediments were closely related to *Bacillus* sp. Nj-19, which was isolated from the Antarctic (Branch 1 in Figure 3; detail in Figure S1). Twenty-two *Flavobacterium* strains recovered from 5 sediments were closely related to *Flavobacterium degerlachei* strain LMG 21915 from microbial mats in Antarctic lakes [13] and strain KOPRI_22212 from the Arctic Ocean (Branch 3 in Figure 3; detail in Figure S3). The phylogenetic relationships of the other strains to their closely related species are also shown in Figure 3. Several strains, such as SS9.17, SS11.5 (Bacteroidetes) and SS13.21 (Actinobacteria), exhibited a distant relationship to the previously identified species. They may represent potentially new species. Furthermore, strains SS9.12, SS9.38, SS14.29, SS14.30 and SS14.31 clustered together and had a very distant relationship with other known Bacteroidetes species, suggesting that these strains may affiliate with a new genus. 

### Diversity of bacterial extracellular proteases in the sediment samples

The diversity of the bacterial extracellular proteases in the sediments was investigated using the protease inhibitors assay (Table 2). PMSF (serine protease inhibitor), OP (metalloprotease inhibitor), E-64 (cysteine protease inhibitor) and Pepstatin A (aspartic protease inhibitor) were used to inhibit the activities of the proteases secreted by the screened strains for identification of these proteases. When the 105 strains were cultured in the liquid screening medium, as many as 85 strains did not produce enough proteases for inhibition analysis. Similar difficulties applying this screening technique to Antarctic bacteria have been previously described [9]. Therefore, only 20 strains were subjected to inhibition analysis. PMSF inhibited the activities of the proteases from 17 strains by 20-100%, showing that almost all the strains produce serine proteases in varying proportions. Among these 17 strains, the activities of the proteases from 12 strains were inhibited by PMSF by more than 90%, which indicated that these strains produce mainly or exclusively serine proteases. Of all 20 strains, OP inhibited the activities of the proteases from 7 strains by 20-100% and had little or no inhibitory effect on the others, showing that the 7 inhibited strains produce metalloproteases. In contrast, E-64 and pepstatin A had less than 10% or no inhibitory effect on the protease activities of all the 20 strains, indicating that these strains hardly produce cysteine or aspartic proteases. Therefore, nearly all the extracellular proteases detected from these bacteria in the sediment samples were serine proteases or metalloproteases. 

**Table 2:** Effects of inhibitors on the extracellular proteases produced by the strains screened from sediments from Maxwell Bay, King George Island.

Class	Genera	Strains	Inhibition Ratio^a^ (%)			
			PMSF^b^(1 mM)	O-P^c^(1 mM)	P-A^d^(0.1 mM)	E64(0.1 mM)
Actinobacteria	*Micrococcus*	SS9.41	99.29	8.78	-3.85	-2.59
	*Micrococcus*	SS14.9	91.56	24.85	7.96	8.56
	*Micrococcus*	SS14.11	102.03	4.95	10.94	-2.07
	*Micrococcus*	SS15.24	99.64	4.16	-2.83	-1.20
Bacilli	*Bacillus*	SS14.37	-2.44	97.39	-0.34	2.06
	*Bacillus*	SS15.18	80.41	20.98	2.18	0.65
	*Planococcus*	SS9.30	26.58	80.85	7.43	0.63
Flavobacteria	*Flavobacterium*	SS9.1	95.19	12.81	8.89	8.94
	*Flavobacterium*	SS9.2	3.89	102.12	5.03	6.09
	*Flavobacterium*	SS9.7	96.08	-3.12	-3.33	-1.76
	*Flavobacterium*	SS9.25	97.27	13.87	6.88	4.62
	*Flavobacterium*	SS9.33	101.23	16.22	6.86	0.61
	*Flavobacterium*	SS14.4	99.57	-2.86	-3.75	-4.64
	unknown	SS9.38	-4.86	84.62	-2.49	-2.13
	unknown	SS14.31	23.86	93.27	7.27	2.03
γ-Proteo^e^	*Pseudoalteromonas*	SS7.1	78.06	17.97	3.22	5.30
	*Pseudoalteromonas*	SS7.3	93.75	5.70	4.37	-1.66
	*Pseudoalteromonas*	SS7.9	87.47	18.07	2.05	-2.38
	*Pseudoalteromonas*	SS7.11	99.90	5.11	-1.17	3.23
	*Pseudoalteromonas*	SS8.3	100.08	4.69	-3.09	-4.69

a The activity of a sample without any inhibitor was taken as control (100%). The inhibition ratio was taken as the result of control activity minus the relative activity of a sample with an inhibitor

b Phenylmethylsulfonyl fluoride

c 1,10-Phenanthroline

d Pepstatin A

e Gammaproteobacteria

Furthermore, the extracellular protease activity of the 105 strains was also investigated by measuring the H/C ratio on the casein and gelatin containing plates. Of the 105 strains, only 57 showed an obvious hydrolytic zone around a single colony, as shown in Table 3 (values >1.1). The H/C ratios of these 57 strains showed considerable variability. The extracellular proteases from strains SS9.41, SS10.34, SS14.19 and SS14.26 had H/C ratios greater than 8, indicating that these strains may have high extracellular protease-producing ability or that their extracellular proteases had high specificity toward casein and gelatin. In contrast, the other 48 strains only showed a hydrolytic zone around a cluster of colonies, but no obvious hydrolytic zone around a single colony, indicating the low ability of these strains to produce extracellular proteases or the low specificity of their proteases toward casein and gelatin.

**Table 3:** H/C ratio of the strains screened from sediments from Maxwell Bay, King George Island on casein-gelatin plates.

Class	Strains	H/C ratio^a^	Strains	H/C ratio^a^
Actinobacteria	*Arthrobacter* sp. SS9.6	1.1^b^	*Arthrobacter* sp. SS13.34	1.1
	*Janibacter* sp. SS14.10	3.46	*Leifsonia* sp. SS15.19	1.1
	*Microbacterium* sp. SS10.1	2.5	***Micrococcus* sp. SS9.41**	**8.57**
	*Micrococcus* sp. SS13.21	1.1	*Micrococcus* sp. SS13.30	1.1
	***Micrococcus* sp. SS14.9**	**1.1**	***Micrococcus* sp. SS14.11**	**1.1**
	***Micrococcus* sp. SS15.24**	**1.1**	*Nocardiopsis* sp. SS15.23	1.6
	*Streptomyces* sp. SS13.32	2.33		
Bacilli	*Bacillus* sp. SS7.7	1.1	*Bacillus* sp. SS8.1	1.1
	*Bacillus* sp. SS8.4	1.1	*Bacillus* sp. SS8.5	3
	*Bacillus* sp. SS9.13	1.1	*Bacillus* sp. SS9.15	1.1
	*Bacillus* sp. SS10.32	4.17	*Bacillus* sp. SS10.33	5
	*Bacillus* sp. SS10.37	10	*Bacillus* sp. SS14.5	1.1
	*Bacillus* sp. SS14.19	8.33	*Bacillus* sp. SS14.20	1.1
	*Bacillus* sp. SS14.21	1.1	*Bacillus* sp. SS14.22	1.1
	*Bacillus* sp. SS14.33	6	*Bacillus* sp. SS14.35	1.5
	***Bacillus* sp. SS14.37**	**1.1**	*Bacillus* sp. SS15.1	1.1
	*Bacillus* sp. SS15.14	2.73	*Bacillus* sp. SS15.15	1.1
	*Bacillus* sp. SS15.16	1.1	***Bacillus* sp. SS15.18**	**1.1**
	*Bacillus* sp. SS15.20	1.1	*Bacillus* sp. SS15.21	1.1
	*Exiguobacterium* sp. SS9.34	5.5	***Planococcus* sp. SS9.30**	**1.1**
Flavobacteria	*Aequorivita* sp. SS14.16	6	***Flavobacterium* sp. SS9.1**	**1.18**
	***Flavobacterium* sp. SS9.2**	**5**	***Flavobacterium* sp. SS9.7**	**1.5**
	*Flavobacterium* sp. SS9.11	1.1	***Flavobacterium* sp. SS9.25**	**1.38**
	*Flavobacterium* sp. SS9.28	1.25	***Flavobacterium* sp. SS9.33**	**1.1**
	*Flavobacterium* sp. SS9.40	1.67	*Flavobacterium* sp. SS10.14	1.54
	*Flavobacterium* sp. SS10.31	3.33	*Flavobacterium* sp. SS11.4	1.5
	*Flavobacterium* sp. SS11.5	2.86	*Flavobacterium* sp. SS11.6	1.5
	*Flavobacterium* sp. SS11.9	1.78	*Flavobacterium* sp. SS11.10	1.1
	*Flavobacterium* sp. SS11.11	6. 67	*Flavobacterium* sp. SS11.13	1.54
	*Flavobacterium* sp. SS11.16	1.1	***Flavobacterium* sp. SS14.4**	**5.33**
	*Flavobacterium* sp. SS15.22	4.62	*Flavobacterium* sp. SS15.25	1.1
	*Flavobacterium* sp. SS15.33	1.5	*Formosa* sp. SS13.7	1.33
	*Formosa* sp. SS15.5	1.5	*Formosa* sp. SS15.6	1.2
	*Gillisia* sp. SS10.34	12.5	*Gillisia* sp. SS10.36	5.25
	*Lacinutrix* sp. SS7.4	1.1	*Lacinutrix* sp. SS7.5	1.1
	*Lacinutrix* sp. SS7.8	1.1	*Lacinutrix* sp. SS7.15	3.5
	*Lacinutrix* sp. SS9.3	1.1	*Lacinutrix* sp. SS9.4	1.2
	*Lacinutrix* sp. SS9.35	1.5	*Lacinutrix* sp. SS10.11	3.75
	*Lacinutrix* sp. SS11.14	5	*Lacinutrix* sp. SS11.19	4.55
	*Lacinutrix* sp. SS13.6	1.1	*Lacinutrix* sp. SS13.13	1.1
	*Lacinutrix* sp. SS13.19	1.1	*Lacinutrix* sp. SS13.20	1.1
	*Lacinutrix* sp. SS13.25	2.2	*Lacinutrix* sp. SS15.30	1.5
	*Lacinutrix* sp. SS15.31	1.92		
	*Psychroserpens* sp. SS9.5	3	*Psychroserpens* sp. SS15.27	3.33
Unknown Bacteroidetes	bacterium strain SS9.12	1.1	bacterium strain SS9.17	1.1
	**bacterium strain SS9.38**	**1.1**	bacterium strain SS14.29	1.1
	bacterium strain SS14.30	1.1	**bacterium strain SS14.31**	**10**
	*Zobellia* sp. SS15.26	1.1		
α-Proteo^c^	*Caulobacter* sp. SS14.14	1.1	*Hyphomonas* sp. SS14.26	10
	*Sphingopyxis* sp. SS10.35	1.1		
β-Proteo^d^	*Burkholderia* sp. SS11.12	7.67		
γ-Proteo^e^	*Photobacterium* sp. SS13.1	1.1	***Pseudoalteromonas* sp. SS7.1**	**7**
	***Pseudoalteromonas* sp. SS7.3**	**3**	***Pseudoalteromonas* sp. SS7.9**	**2.08**
	*Pseudoalteromonas* sp. SS7.10	2.5	***Pseudoalteromonas* sp. SS7.11**	**3.67**
	*Pseudoalteromonas* sp. SS7.12	3.67	***Pseudoalteromonas* sp. SS8.3**	**1.1**

The 20 strains listed in Table 2 were highlighted by bold type.

a H/C ratio is the ratio of the hydrolytic zone diameter to the colony diameter of a colony on the plate

b 1.1 represents a slight hydrolytic zone formed by the strain

c Alphaproteobacteria

d Betaproteobacteria

e Gammaproteobacteria

## Discussion

Investigating the diversity of bacteria is critical to understanding how global biogeochemical cycles function. In this study, we investigated the protease-producing bacterial communities and the diversity of extracellular proteases produced by these bacteria in Antarctic coastal sediments.

In general, the cultivability (cultivable population vs. total cell population) of the marine microbial community is less than 1% [14]. The richness of the cultivable protease-producing bacteria in 8 sediments of Maxwell Bay studied in this paper was ~10^5^ cells/g in all sediments, indicating the existence of a sizable population of cultivable bacteria in the sediments. However, this finding is an order of magnitude lower than the richness of cultivable protease-producing bacteria in the South China Sea (~10^6^ cells/g) [3]. Because the levels of OrgC and OrgN in the collected Antarctic samples are not lower but rather higher than those in the samples collected from the South China Sea, the low richness of cultivable protease-producing bacteria in the Antarctic sediments may be due to the low growth rate of the polar bacteria in a permanently cold condition, which merits further study.

Bacteroidetes are an important bacterial group in high-latitude marine sediments. Actinobacteria make up only a minor fraction of microbial communities in marine sediment samples [15-17]. Loperena et al. recovered some protease-producing *Arthrobacter* strains affiliated with Actinobacteria from the marine sediment next to the Artigas Base on King George Island [18]. Our results showed that the protease-producing bacterial strains were affiliated with the Bacteroidetes, Proteobacteria, Actinobacteria and Firmicutes phyla. Strains in the *Flavobacterium*, *Lacinutrix* and *Bacillus* genera were the most cultivated, accounting for 60% of all strains, while only 8 Gammaproteobacteria strains were recovered. Although *Bacillus* was the predominant genus in the recovered protease-producing strains from Antarctic coastal sediments, their protease-producing ability was generally low based on the analyses of their H/C ratio on plates and their protease activity in liquid culture. Previous studies showed that Gammaproteobacteria are an important population in marine sediments [19-21], and are also the main protease-producing bacteria [2,3]. Though only 8 *Pseudoalteromonas* strains were recovered, 6 of these strains had an H/C ratio ˃ 2, and 5 could produce enough enzymes in liquid culture to perform the inhibitor assay, indicating that the *Pseudoalteromonas* strains in Antarctic coastal sediments may have a high protease-producing ability in general. This corroborates previous work by Xiong et al. that found *Pseudoalteromonas* strains showed high protease-producing ability in deep-sea sediments from near the Arctic [22]. In addition, Flavobacteria also displayed a good protease-producing ability in general, according to their H/C ratio on plates and their protease activity in liquid culture. 

Among the protease-producing strains recovered from Antarctic coastal sediments, only approximately 54% (57/105) could form an obvious hydrolytic zone around a colony on plates, and only 20% (20/105) of strains produced enough enzymes for inhibitor assays in liquid culture. In contrast, 87% (68/78) of bacterial strains screened from the sediment in the South China Sea could form an obvious hydrolytic zone around a colony on plates, and 80% (62/78) could produce enough enzymes for inhibitor assays [3]. Therefore, it seems that the protease-producing ability of the bacteria recovered from Antarctic coastal sediments is generally lower than that of the bacteria recovered from South China Sea sediments. 

Vazquez et al. [8] screened 33 protease-producing bacterial isolates from several marine biotopes in King George Island, 5 of which were from marine sediment and 11 of which were from seawater. Among these isolates, the protease produced by strain *Pseudoalteromonas* sp. P96-47 from seawater was found to be a metalloprotease [8]. Our results showed that nearly all the extracellular proteases produced by the screened sedimentary bacteria are serine proteases or metalloproteases, similar to the findings on sediments from the South China Sea. Enzymes secreted by bacteria inhabiting cold sediments are usually cold-active enzymes that exhibit high catalytic efficiency at low and moderate temperatures and are easily inactivated by a moderate increase in temperature [23]. Therefore, some of these newly detected proteases produced by Antarctic marine sediment bacteria may have biotechnological potential. Detailed identification of these proteases will be addressed in a future study. 

## Supporting Information

Figure S1 **The Neighbor-joining phylogenetic tree of the strains in Branch 1 in Figure 3 based on the 16S rDNA sequences.**
(TIF)

Figure S2 **The Neighbor-joining phylogenetic tree of the strains in Branch 2 in Figure 3 based on the 16S rDNA sequences.**
(TIF)

Figure S3 **The Neighbor-joining phylogenetic tree of the strains in Branch 3 in Figure 3 based on the 16S rDNA sequences.**
(TIF)

Figure S4 **The Neighbor-joining phylogenetic tree of the strains in Branch 4 in Figure 3 based on the 16S rDNA sequences.**
(TIF)

Figure S5 **The Neighbor-joining phylogenetic tree of the strains in Branch 5 in Figure 3 based on the 16S rDNA sequences.**
(TIF)
