# Supplementary figures and images for: Diversity of Both the Cultivable Protease-Producing Bacteria and Bacterial Extracellular Proteases in the Coastal Sediments of King George Island, Antarctica
Source: PLoS One. 2013 Nov 4;8(11):e79668. doi: 10.1371/journal.pone.0079668 (PMC3817139; doi:10.1371/journal.pone.0079668)

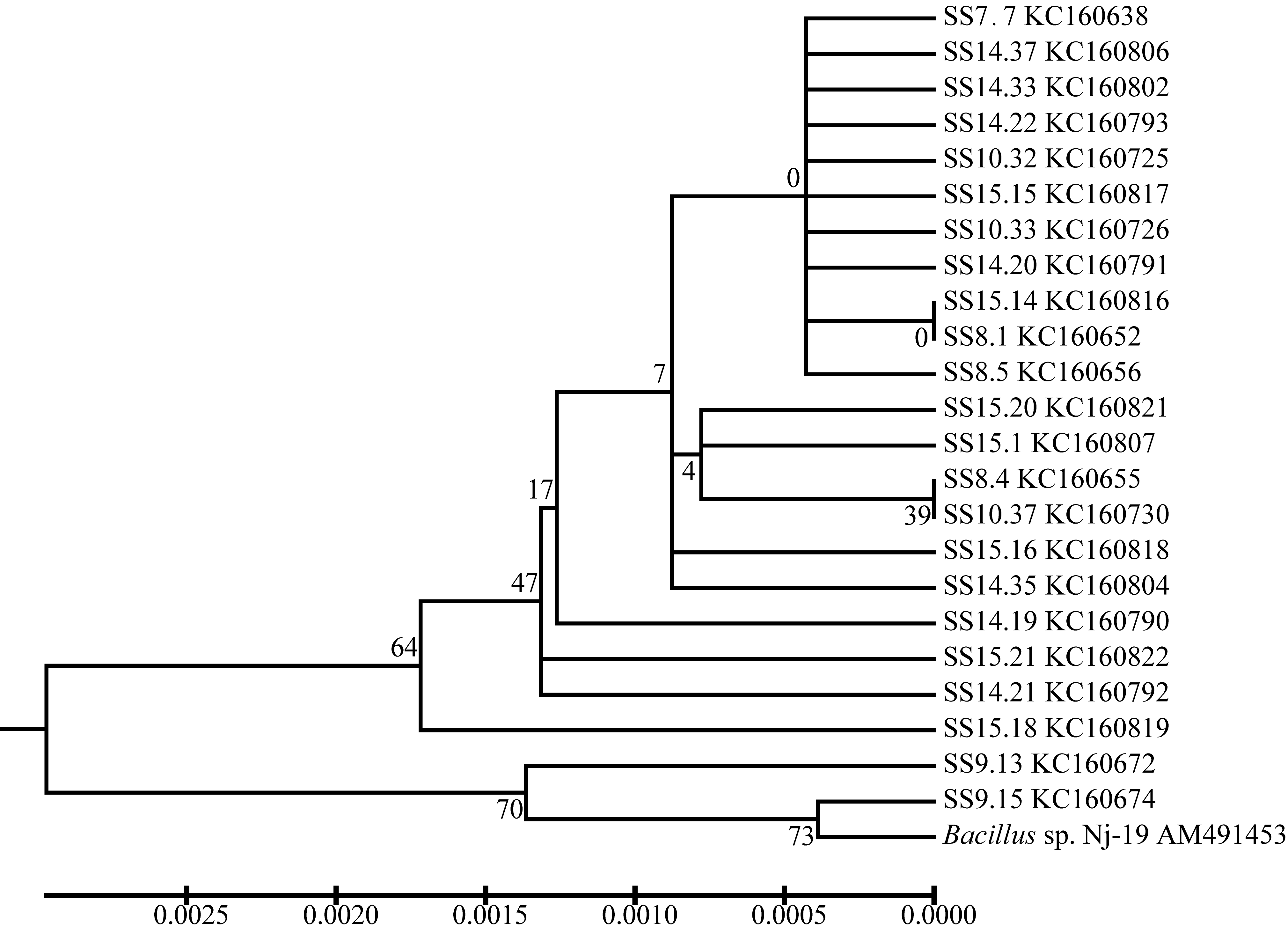

Supplement: Figure S1 — The Neighbor-joining phylogenetic tree of the strains in Branch 1 in Figure 3 based on the 16S rDNA sequences. (TIF) [file pone.0079668.s001.tif]

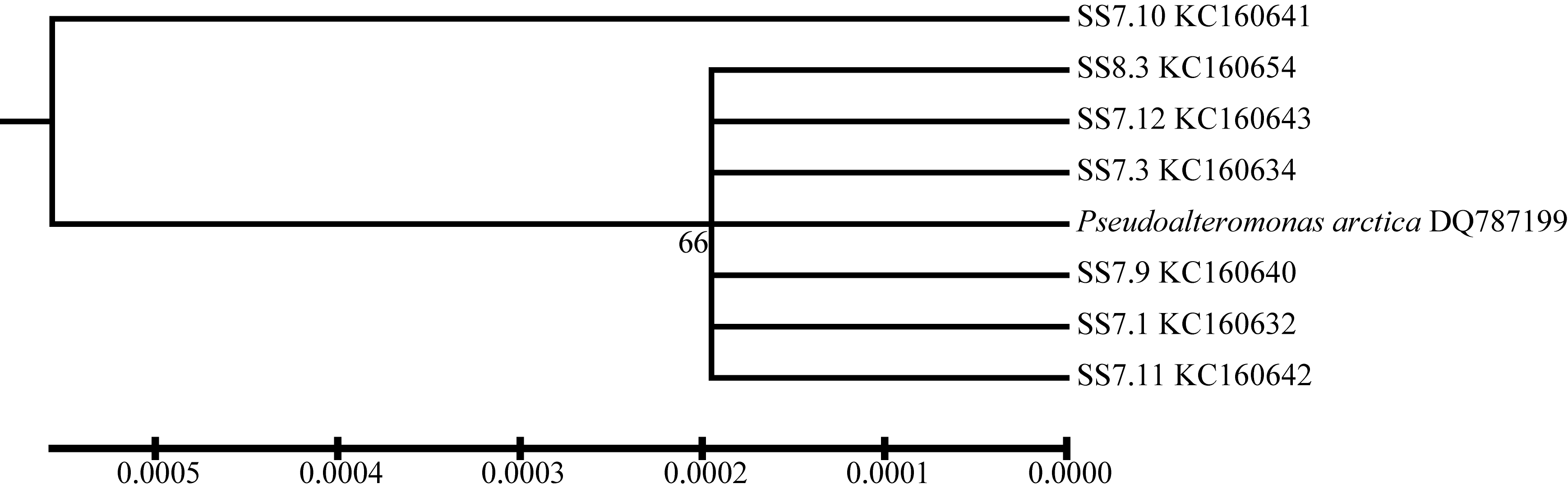

Supplement: Figure S2 — The Neighbor-joining phylogenetic tree of the strains in Branch 2 in Figure 3 based on the 16S rDNA sequences. (TIF) [file pone.0079668.s002.tif]

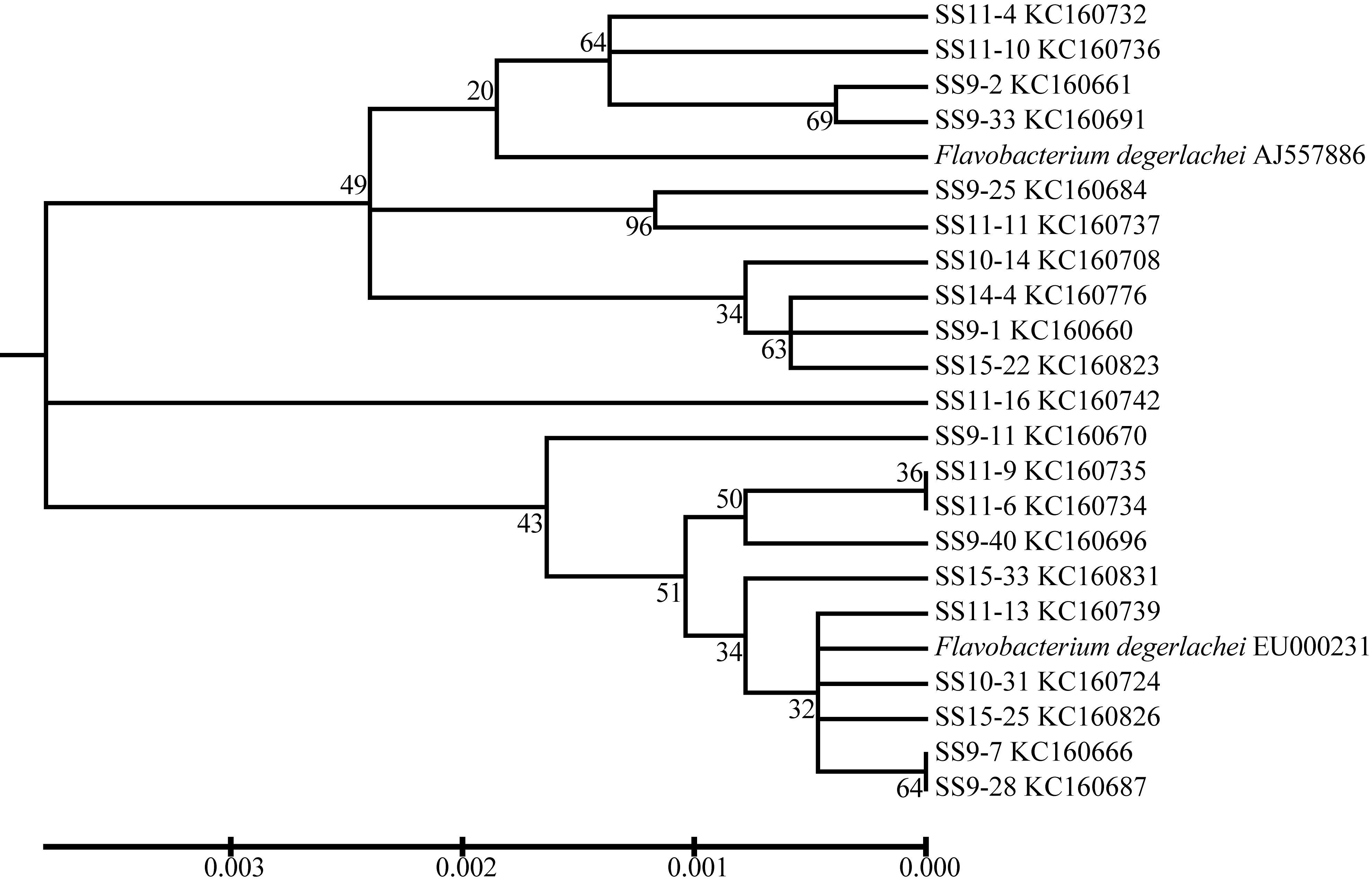

Supplement: Figure S3 — The Neighbor-joining phylogenetic tree of the strains in Branch 3 in Figure 3 based on the 16S rDNA sequences. (TIF) [file pone.0079668.s003.tif]

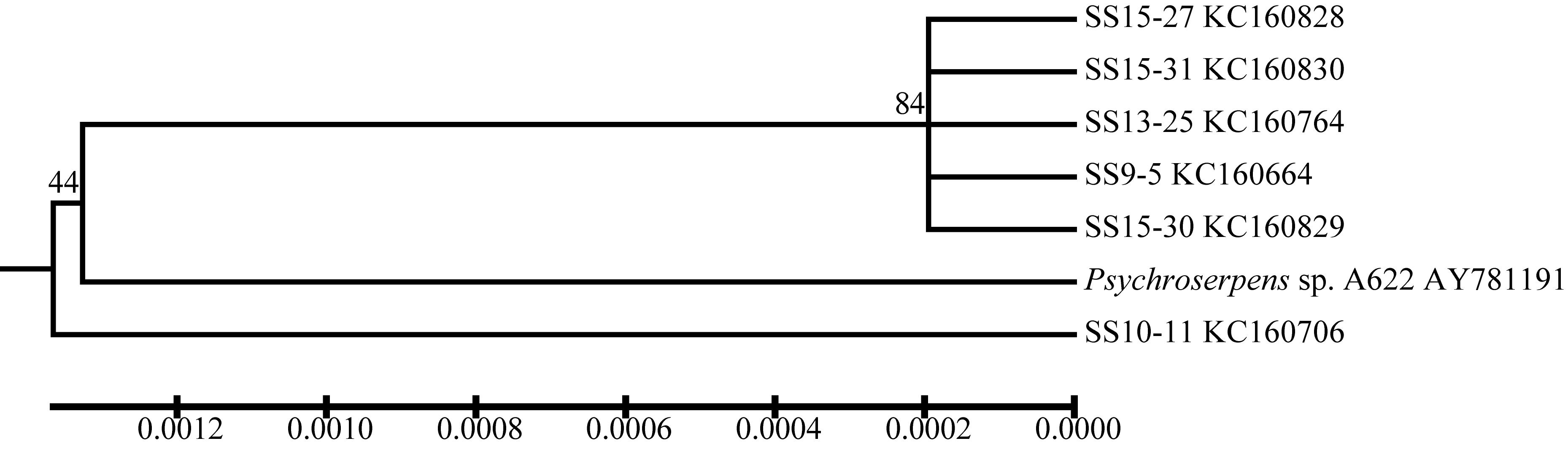

Supplement: Figure S4 — The Neighbor-joining phylogenetic tree of the strains in Branch 4 in Figure 3 based on the 16S rDNA sequences. (TIF) [file pone.0079668.s004.tif]

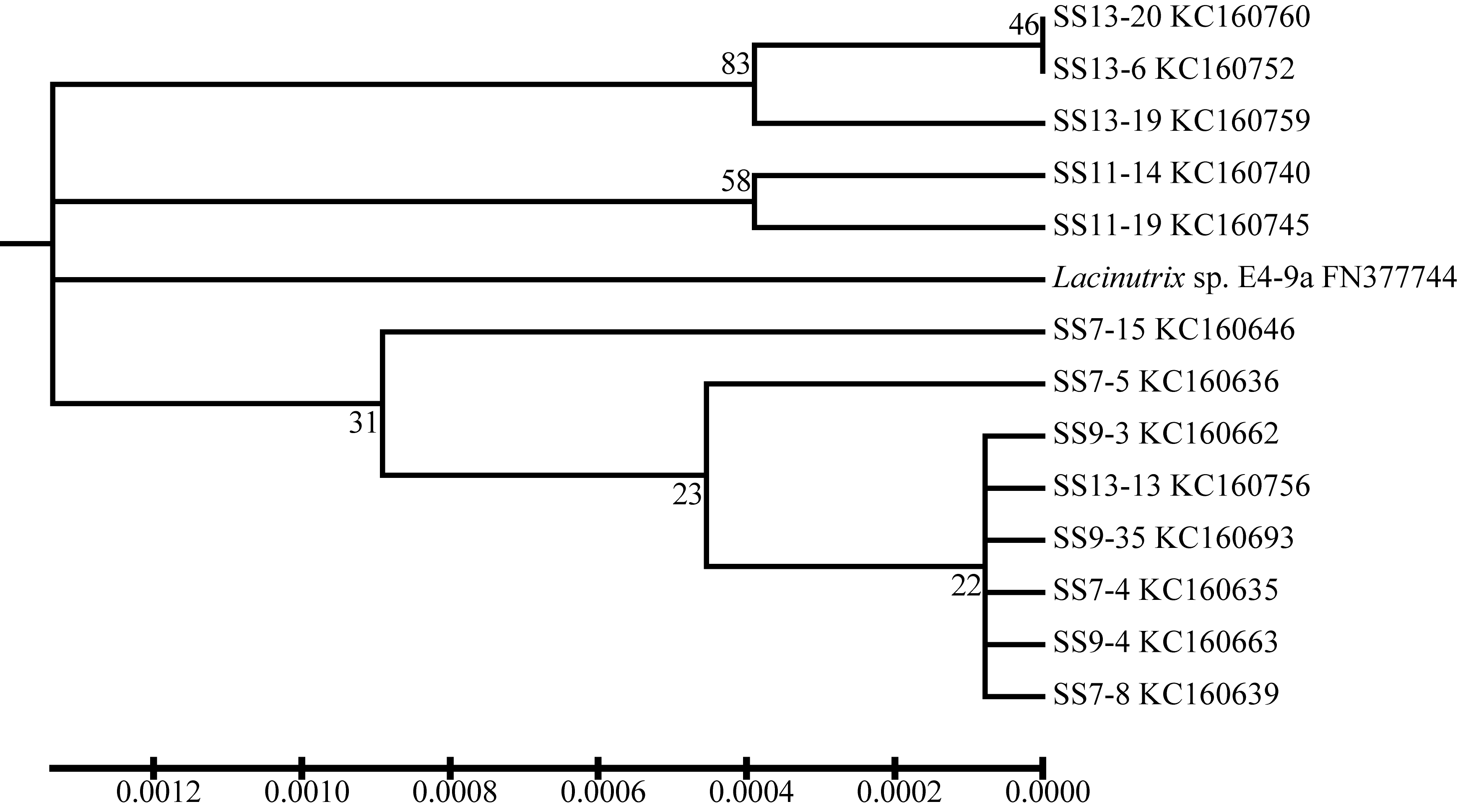

Supplement: Figure S5 — The Neighbor-joining phylogenetic tree of the strains in Branch 5 in Figure 3 based on the 16S rDNA sequences. (TIF) [file pone.0079668.s005.tif]
